# Predominantly Orphan Secretome in the Lung Pathogen *Mycobacterium abscessus* Revealed by a Multipronged Growth-Phase-Driven Strategy

**DOI:** 10.3390/microorganisms12020378

**Published:** 2024-02-12

**Authors:** Harish Chandra, Manish K. Gupta, Ying-Wai Lam, Jagjit S. Yadav

**Affiliations:** 1 Pulmonary/Microbial Pathogenesis Laboratory, Department of Environmental and Public Health Sciences, University of Cincinnati College of Medicine, Cincinnati, OH 45267, USA; chandrhh@ucmail.uc.edu (H.C.);; 2 Vermont Biomedical Research Network Proteomics Facility, University of Vermont, Burlington, VT 05405, USA

**Keywords:** *Mycobacterium abscessus*, secretome, bottom-up proteomics, lung infections, hypersensitivity pneumonitis, metalworking fluid

## Abstract

The emerging lung pathogen *Mycobacterium abscessus* is understudied for its virulence determinants and molecular targets for diagnosis and therapeutics. Here, we report a comprehensive secretome (600 proteins) of this species, which was identified using a multipronged strategy based on genetic/genomic, proteomic, and bioinformatic approaches. In-solution digested bottom-up proteomics from various growth phases identified a total of 517 proteins, while 2D-GE proteomics identified 33 proteins. A reporter-gene-fusion-based genomic library that was custom-generated in this study enabled the detection of 23 secretory proteins. A genome-wide survey for N-terminal signal sequences using bioinformatic tools (Psortb 2.0 and SignalP 3.0) combined with a strategy of the subtraction of lipoproteins and proteins containing multiple transmembrane domains yielded 116 secretory proteins. A homology search against the *M. tuberculosis* database identified nine additional secretory protein homologs that lacked a secretory signal sequence. Considering the little overlap (80 proteins) among the different approaches used, this study emphasized the importance of using a multipronged strategy for a comprehensive understanding of the secretome. Notably, the majority of the secreted proteins identified (over 50%) turned out to be “orphans” (those with no known functional homologs). The revelation of these species-specific orphan proteins offers a hitherto unexplored repertoire of potential targets for diagnostic, therapeutic, and vaccine research in this emerging lung pathogen.

## 1. Introduction

Non-tuberculous mycobacteria (NTM) have been known to cause various diseases in both immunosuppressed and immunocompetent individuals [1]. In particular, species of the *Mycobacterium chelonae*—*Mycobacterium abscessus* (MCA) complex belonging to the rapidly growing mycobacteria (RGM) group of NTM are associated with both infectious and immune-mediated pulmonary diseases and nosocomial infections [2]. *Mycobacterium abscessus*, a key member of this complex, was recovered from human skin and soft tissue infections more than 50 years ago [3]. *M. abscessus* accounts for more than 80% of all pulmonary infections caused by the RGM species of NTM [4]. *M. abscessus* infection has been on the rise, especially in patients with underlying chronic lung diseases, such as bronchiectasis, COPD, and cystic fibrosis [5].

*M. abscessus* and other species of the MCA complex are prevalent in aqueous and terrestrial environmental niches. Notably, they are known to colonize industrial water-based metalworking fluids (MWFs) and are considered etiological agents of an immune-mediated lung disease, hypersensitivity pneumonitis (HP), in machine workers exposed to contaminated MWF aerosols [6,7]. One of the reasons for the selective colonization of harsh aqueous environments, including MWF, by the MCA complex, including this species, is their greater resistance to biocides and chlorine disinfectants than that of *M. tuberculosis* and other virulent species of mycobacteria [8]. In the context of antimicrobial resistance, *M. abscessus* is considered the most antibiotic-resistant NTM species and, therefore, poses a serious threat to human health [1]. Antimycobacterial therapy for *M. abscessus* infection is difficult, as most common frontline mycobacterial drugs are relatively ineffective, and mono-drug treatments are not effective.

*M. abscessus*, which causes tuberculous-like lesions in human infections, has been shown to persist for up to 45 days in soft tissues, such as the kidney and liver, in mouse infection models, and it forms typical granuloma-like structures [9,10]. In pathogenic mycobacteria, virulence factors are critical in host pathogenesis and immunity and offer a potential for use in the development of diagnostics, vaccines, and therapeutics [11,12]. Unlike the pathogenic species of slowly growing mycobacteria (SGM) such as *M. tuberculosis*, *M. bovis*, *M. leprae*, and *M. avium*, little is known about the virulence factors of rapidly growing mycobacteria (RPM) species in general and species of the *M. chelonae*–*M. abscessus* complex in particular. While the whole genome sequence of *M. abscessus* has been reported [13], there is a knowledge gap concerning the specific virulence factors. In this context, secreted proteins are prominent among the known specific virulence factors in pathogenic mycobacteria and, particularly, *M. tuberculosis.* The profiling of the secretory proteins of a pathogen can, therefore, reveal its potential virulence and antigenicity factors [14]. Several secreted proteins from the culture filtrates of tuberculous mycobacterial species have been reported, and they have been characterized as effectors in terms of transportation and secretion mechanisms and their roles in the antigenicity or virulence of those organisms [15]. Such studies on nontuberculous mycobacteria (NTM) species, including *M. abscessus*, are lacking. Considering this rationale, there is a pressing need to fully understand the secretome in *M. abscessus*. The aim of this study was, therefore, to identify the secretory protein repertoire of *M. abscessus*, the most pathogenic and drug-resistant MCA species with a poorly understood virulome.

Preliminary efforts by us and others using 2D-proteomic analysis pointed to the presence of species-specific proteins in this organism using genomically uncharacterized strains [14,16,17]. In this direction, our efforts on *M. abscessus* indicated species-specific secretome differences from the closely related species *M. chelonae* [17]. Similar initial efforts showed differences between *M. abscessus* and another related species, *M. massiliense* [16]. Therefore, the comprehensive experimental revelation of a genome-wide secretory protein repertoire by using the genome strain was necessitated. In terms of computational studies, there are few reports on the prediction of the mycobacterial secretome using pre-existing algorithms [18]. The major drawback of these bioinformatic platforms is that they fail to predict secreted proteins that lack a signal peptide. Also, these bioinformatic-based pipelines require validation using experimental approaches [19]. Considering that no single approach has been successful in yielding all candidate secreted proteins in mycobacterial organisms, including *M. abscessus*, we employed a multipronged strategy based on reporter gene fusion, bioinformatic, and proteomic approaches. Such comprehensive identification of species-specific secreted proteins in *M. abscessus* could help distinguish it from other closely related species of the *M. chelonae*–*M. abscessus* group, as well as from other groups of pathogenic mycobacteria, and it could allow them to serve as potential diagnostic and/or therapeutic targets for the treatment of infections with this organism [20]. To our knowledge, this is the first systematic multi-pronged attempt at the genome-wide identification of the secreted proteins (secretome) in *M. abscessus* using such a combination of experimental and computational approaches. The characterization of such a comprehensive secretome enabled the identification of several candidate virulence effectors based on known functional homologs in pathogenic mycobacteria of the *M. tuberculosis* complex and revealed a large number of novel ‘hypothetical’ proteins that indicated a predominantly “orphan” secretome.

## 2. Materials and Methods

### 2.1. Strains and Plasmids

*Mycobacterium abscessus* strain ATCC 19977 was obtained from the American Type Culture Collection (ATCC), maintained on Middlebrook 7H10 (MB7H10) agar, and grown in Sauton’s medium. Signal sequence capturing vector pJEM11 was a gift from Gicquel Laboratory at the Pasteur Institute, Paris, France [21]. This vector has alkaline phosphatase as a reporter gene lacking a signal sequence.

### 2.2. In Silico Genome-Wide Identification of the Secreted Proteins of M. abscessus

A total of 4920 proteins were predicted in the *M. abscessus* genome using the NCBI genome database [22]. Secretory protein sequences were analyzed in the whole genome using the Psortb version 2.0 and SignalP version 3.0 software from Biohealthbase–Mycobacterial–protein localization (http://www.psort.org/psortb/ (accessed on 17 January 2024), http://www.cbs.dtu.dk/services/SignalP/ (accessed on 17 January 2024)), which were available online. The putative secretory proteins predicted in silico were then scanned with the DeepTMHMM version 1.0.18 (https://dtu.biolib.com/DeepTMHMM (accessed on 17 January 2024)) software for the presence of transmembrane domains other than the N-terminal signal sequence. The proteins showing no repetitive transmembrane domains other than N-terminal signal sequences were scanned to eliminate lipoproteins using the LipoP version 1.0 and LipPred (http://www.cbs.dtu.dk/services/LipoP/ (accessed on 17 January 2024; http://www.ddg-pharmfac.net/lippred/LipPred/lippred.htm (accessed on 17 January 2024)) software. The resulting protein candidates were categorized as true secretory proteins. These secreted proteins that were identified in silico were annotated to identify homologs of proteins of known functions and hypothetical proteins of unknown functions. The predicted proteins were then categorized based on their biological functions. Signal-sequence-lacking secretory proteins were also searched in the *M. abscessus* genome database using a literature search and BLAST search against the *M. tuberculosis* secretory proteins.

### 2.3. Construction of the M. abscessus Genomic Library

The genomic DNA of *M. abscessus* was isolated from a log phase culture using the CTAB method [23]. The DNA was digested with Sau3A (NEB, Ipswich, MA, USA) and run on 1.5% agarose gel. DNA fragments ranging from 200 to 1500 bp in size were eluted using a gel elution kit (Qiagen, Valencia, CA, USA). The eluted DNA was ligated into the pJEM11 vector, predigested with BamH1, and dephosphorylated using calf intestinal phosphatase (NEB, Ipswich, MA, USA) with an insert-to-vector ratio of 2:1. A ligation mixture was used to transform the chemically competent *E. coli* DH5α cells and the selected transformants on a Luria Bertani (LB) agar plate containing kanamycin (50 μg/mL).

### 2.4. Construction of Genomic phoA Fusion Library for M. smegmatis and Isolation of phoA+ Transformants

Approximately 10,000 individual genomic clones were selected and pooled for isolation of their recombinant plasmids. The pooled plasmid DNA was electroporated into *M. smegmatis* MC2, and the transformants were selected on Middlebrook 7H10 agar (with 10% OADC) plates containing kanamycin (20 μg/mL) and 5-bromo-4-chloro-3-indolyl phosphate (BCIP; 60 μg/mL) by incubating at 37 °C for a week. Beginning on day 3, the plates were observed daily for bacterial growth. Colonies showing a blue color due to the expressed alkaline phosphatase activity (PhoA) on BCIP were selected for further analysis.

### 2.5. Measurement of the Phosphatase Activity of Recombinant M. smegmatis

The alkaline phosphatase (PhoA) activity of *M. smegmatis* clones grown in liquid culture was measured as described previously [24]. The PhoA activity was measured in whole-cell lysates by mixing 0.1 mL of the sample and 1 mL of detection buffer (1 M Tris-HCl pH 8.0, 4 mM p-nitrophenyl phosphate [pNPP]). The reaction was stopped after 40 min of incubation at 37 °C using 100 µL of 1M K_2_HPO_4_. The reaction mixture was centrifuged for 10 min at maximum speed using a microcentrifuge, and the activity in the supernatant was measured at 420 nm. *M. smegmatis MC2* pJEM11 was used as a negative control in these assays.

### 2.6. DNA Sequencing and Sequence Analysis

Plasmid DNA isolated from the positive *M. smegmatis* clones (the blue colonies) propagated in *E. coli* DH5α was used to identify the insert DNA (signal sequence). The presence of the DNA insert was first checked through restriction digestion. Inserts in the confirmed clones were sequenced directly in the pJEM11 plasmids using vector-specific primers (forward 5′ CTAGTACTGGGCCCGCGGAT 3′; reverse 5′ CCCCATCCCATCGCCAAT 3′). The insert DNA sequences were then searched against the *M. abscessus* genome database (https://blast.ncbi.nlm.nih.gov/Blast.cgi?PROGRAM=blastn&PAGE_TYPE=BlastSearch&BLAST_SPEC=MicrobialGenomes_561007&LINK_LOC=blasttab&PROG_DEFAULTS=on&SEARCH_INIT=ReprGenomeDBSearch&TAXID=561007&LAST_PAGE=tblastn (accessed on 23 November 2022)).

### 2.7. Preparation of Culture Filtrate Proteins

*M. abscessus* cells grown to different stages of growth (4 replicates each), including five minutes post-inoculation incubation only (“5 min”), early-log (EL) phase (30 Klett reading), mid-log (ML) phase (175 Klett reading), and late-log (LL) phase (325 Klett readings), were pelleted via centrifugation at 8000 rpm (20 min, 4 °C). The resulting culture supernatant was filtered (0.22 microns) to remove any remaining intact cells. The resulting culture filtrate was subjected to protein precipitation using the trichloroacetic acid method [14], and the protein precipitate was washed thrice with acetone. The sterile medium was processed in parallel for the generation of a negative control. The dried protein pellets were subjected to in-solution-digested shotgun proteomics analysis, as described in the subsequent paragraphs. In a separate experiment, dried pellets from the mid-log phase and late-log phase were suspended in a sample preparation solution (8 M urea, 4% CHAPS, 40 mM DTT) for two-dimensional gel electrophoresis analysis. Based on the protein estimation with the DC assay (Bio rad), a 100 μg aliquot of the protein preparation was used for the analysis.

### 2.8. Two-Dimensional Gel Electrophoresis (2D-GE)

For the step of first-dimensional separation via isoelectric focusing (IEF), the secretory protein mixture was first cleaned up using a 2D clean-up kit (GE healthcare, Piscataway, NJ, USA) to remove any impurities, and the cleaned sample was then suspended in 125 μL of destreaking rehydration solution. The IEF was run using a 7 cm immobiline dry strip with a pH of 4–7 (GE Healthcare, Park Ridge, NJ, USA) on an IPGphore II isoelectric focusing system (GE Healthcare, Park Ridge, NJ, USA). The strip was rehydrated (15 °C for 12 h) using 50 V and further focused using the following sequence of parameters: 100 V for 1 h, 500 V for 2 h, 1000 V for 1 h, 2000 V for 2 h, and, finally, 8000 V for 2 h.

The IEF strip was incubated in equilibration buffer I (6 M urea, 30% glycerol, 0.05 M Tris, pH 8.8, 2% SDS, 0.002% bromophenol blue) containing 135 mM DTT for 15 min and then in equilibration buffer II (6 M urea, 30% glycerol, 0.05 M Tris, pH 8.8, 2% SDS, 0.002% bromophenol blue) containing 135 mM iodoacetamide for another 15 min. The IEF-resolved proteins were separated in the second dimension using 12% SDS-PAGE at 70 V. The 2D gels were visualized by staining with SYPRO Ruby (Bio rad). The protein gel spots were picked up using a spot picker and destained for 2–3 h using 50% acetonitrile (ACN)/25 mM NH_4_HCO_3_. After reduction (10 mM DTT) and alkylation with 55 mM iodoacetamide, the gel pieces were washed using 100 mM NH_4_HCO_3_. Following dehydration using ACN and drying in a SpeedVac, these were subjected to trypsin digestion (Promega, Madison, WI, USA) for 18 h at 37 °C. The released peptides were extracted successively with 5% FA/50% ACN and 100% ACN.

### 2.9. Bottom-Up Proteomics

#### 2.9.1. In-Solution Digestion

Trichloroacetic acid (TCA)-precipitated proteins from the four biological replicates of the 5 min, EL, ML, and LL growth phases were suspended in 0.5 mL of 8 M urea/20 mM HEPES (pH 8). The suspensions were sonicated on ice using 3 bursts of 20 s each at 1 min intervals. The protein concentrations in the suspensions were determined with BCA (EL: 0.08 mg/0.5 mL, ML: 0.22 mg/0.5 mL, LL: 1 mg/0.5 mL). Finally, the samples were digested with trypsin with an enzyme-to-substrate ratio of 1:50 after the reduction of disulfides and alkylation. The acidified tryptic peptides were subjected to purification using a Sep-Pak tC18 cartridge (WAT023590, 1cc, 100 mg; Waters, Milford, MA, USA), followed by lyophilization. The dried peptides were kept at −80 °C until LC/MS analysis.

#### 2.9.2. LC/MS, Database Searches, and Data Analysis

The dried digests were re-suspended in 100 μL of a solution of 2.5% acetonitrile (CH_3_CN) and 2.5% formic acid (FA) in water, and 2.5 μL aliquot of the digest was analyzed on a Q-Exactive mass spectrometer coupled with an EASY-nLC (Thermo Fisher Scientific, Waltham, MA, USA) using the standard optimized protocols at the Vermont Biomedical Research Network Proteomics Facility.

#### 2.9.3. Database Searches

Raw files (.raw) were analyzed using Proteome Discoverer 2.5 (Thermo Fisher Scientific). Product ion spectra were searched using SEQUEST with the “Basic” Processing and Consensus workflows against a Uniprot *Mycobacterium abscessus* protein database (UP000007137) based on standard search parameters.

#### 2.9.4. Data Analysis

For spectral counting analysis, the resulting. msf result files were incorporated into Scaffold version 5.1.0 (Proteome Software, Portland, OR, USA) with “prefiltered mode” and “Protein Cluster Analysis”. “Protein Cluster Analysis” was used to group proteins into clusters; one of the cluster members was selected to represent the cluster, and the associated spectral counts were derived from the peptides of all of the members. “No normalization” and a min. value of 0.5 (all spectral counts of 0 were replaced with 0.5) were used. FDR at the protein and peptide levels of 1% and “Min number of peptides” = 2 were selected to achieve a 1.0% protein decoy FDR and 0.14% peptide decoy FDR in the filtered dataset. Spectral counting reports from Scaffold with “Show lower scoring peptides and <5% Probabilities” deactivated and with “Decoys” hidden were exported to an Excel spreadsheet. The exclusive spectrum count for the 5 min, EL, ML, and LL growth phases was statistically evaluated via pairwise comparisons (two-tailed *t*-test). All sorting (using the VLOOKUP function in Excel) and statistical analyses of protein spectral counts were detailed in Excel spreadsheets. Volcano plots were created using fold change (FC) values (log2 of the difference between the number of peptides of the two groups for each protein and −log10 of the *p*-values). Heat maps were created by scaling the spectral counts of individual samples so that all counts from all samples summed up to 100* the number of samples, which allowed the FC values of proteins of varying abundances to be represented on the same scale. Graph Pad Prism 9 (GraphPad Software Inc., San Diego, CA, USA) was used to create the volcano plots and heat maps.

The identified proteins were characterized using the available mycobacterial databases, and the prediction of non-classical protein secretion was performed by SecretomeP version 2.0 server (http://www.cbs.dtu.dk/services/SecretomeP (accessed on 17 January 2024)) and Signal peptide cleavage location was predicated by SignalP version 4.1 server (http://www.cbs.dtu.dk/services/SignalP/ (accessed on 17 January 2024)).

## 3. Results

The growth-phase-specific secretome analysis of *M. abscessus* cultures from the early-log phase to the late-log phase using bottom-up semi-quantitative proteomics comprehensively identified a total of 517 proteins (Table 1, Table 2 and Table 3) and the Supplementary Excel File). In addition, a combination of experimental and computational techniques enabled the identification of a repertoire of 181 secreted proteins in *M. abscessus* ( 4 and 5 and S1), as described in the following sections.

### 3.1. Secretome Identification through Bottom-Up Semi-Quantitative Proteomics

**Figure 1:**
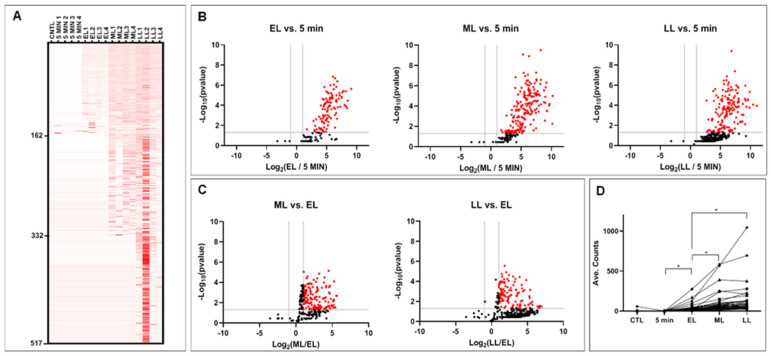
Differential secretory proteomes of *M. abscessus* during various growth phases. Secretory proteins precipitated from supernatants harvested from the control, 5 min, early-log (EL), mid-log (ML), and late-log (LL) cultures of *M. abscessus* were subjected to in-solution digestion and bottom-up proteomic analysis. Differential protein abundances were analyzed via spectral counting. (**A**) Heat map of differential secretory proteomes during various growth phases. Relative abundances (scaled total spectral counts) are shown, with proteins being sorted by *p*-values per the following order: (1) EL vs. 5 min, (2) ML vs. 5 min, and (3) LL vs. 5 min. A total of 517 proteins were identified (<1% false discovery rate) in all growth phases. (**B**) Proteins with an increased abundance in the secretomes of the EL, ML, and LL phases versus the 5 min time point are highlighted in red in the volcano plots. The fold change (FC) value cut-off at 2 (log2 −2 = −1 and log2 2 = 1) and *p*-value cut-off at 0.05 (−log10 0.05 = 1.301) are indicated by a gray line(s) on the *x*-axis and *y*-axis, respectively. (**C**) Proteins that were differentially secreted in the ML phase and LL phase compared to the EL phase. (**D**) Proteins secreted that were during the early phase and continued to be secreted in either the ML or LL phase. A total of 46 differentially expressed proteins that were common to EL vs. 5 min (FC > 2, *: *p* < 0.05), ML vs. EL (FC > 2, *: *p* < 0.05), and LL vs. EL (FC > 2, *: *p* < 0.05) were identified. The numerical data for the plot(s) included in each panel ((**A**) through (**D**)) are provided in the Supplementary Excel File.

To elucidate the dynamics of the secretory proteomic profile of *M. abscessus* during growth, bottom-up semi-quantitative proteomics were conducted on culture supernatants (*n* = 4) collected 5 min post-inoculation and at the early-log (EL) phase, mid-log (ML) phase, and late-log (LL) phase of growth. A culture medium without bacteria was included as a negative control. In total, 517 proteins belonging to 510 clusters were identified (<1% false discovery rate) across the growth phases that were examined (Table 1, Figure 1A, and Supplementary Excel File—“overview_spectrum_counts” sheet, as well as Tables S6 and S7). The lack of detectable proteins in the 5 min control sample indicated that there was no contamination from cellular proteins during the preparation of the secretome samples for analysis. The size of the secretory proteome increased through the growth phases, as evidenced by the identification of 162, 324, and 465 proteins in the EL, ML, and LL phases, respectively; these data based on four replicates showed statistical significance in comparison with the 5 min incubation (FC > 2, *p* < 0.05; Figure 1B, Table 2). There was a significantly different abundance (FC > 2, *p* < 0.05) of secreted proteins in the ML phase (142 proteins) and LL phase (118 proteins) compared to the EL (107 proteins) phase (Figure 1C and Table 2). Common overlapping proteins identified in the different growth phases are shown in Table 3.

The secretory proteomes for the ML and LL phases showed an increasing trend, as proteins were continuously secreted from the EL phase through the ML phase and accumulated in the LL phase (Figure 1B,C). Forty-six proteins were identified as common among the three sets of differentially expressed proteins (FC > 2, *p* < 0.05) identified in EL vs. 5 min, EL vs. ML, and EL vs. LL (Table 3 and Figure 1D). These proteins were secreted during the early phase (FC > 2, *p* < 0.05), and the secretion continued with a significant increase in abundance (FC > 2, *p* < 0.05) in the ML and LL phases compared to that in the EL phase (Figure 1D).

### 3.2. Identification of Secretory Proteins with 2D-GE

**Figure 2:**
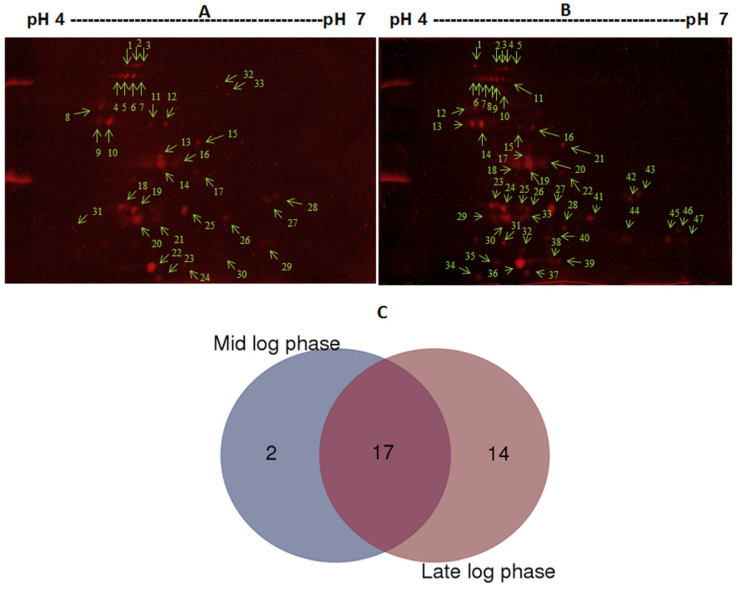
Proteomic profiling of the secretory proteins of *M. abscessus* during the log phases of growth. (Panels **A** and **B**) The 2D-GE profiles of filtrate proteins from cultures grown to the mid-log phase (Panel **A**) and late-log phase (Panel **B**). Individual protein spots (red color) are shown with an arrow and number (green color). The cultures were grown in Sauton’s medium at 37 °C by shaking at 225 rpm for 24 h (180 Klett Reading) and 48 h (400 Klett Reading). (Panel **C**) A Venn diagram showing the differential and overlapping distributions of the secreted proteins between the mid-log and late-log phases.

The independent 2D-GE proteomic analysis of the culture filtrates of actively growing cultures (ML phase and LL phase) combined with mass spectrometry (Figure 2A,B and Table 4, Tables S2 and S3) yielded a total of 33 secreted proteins, with some having multiple occurrences (1–8), suggesting the presence of isotypes (Table 4). Seventeen proteins were found to be common between the ML phase and LL phase, whereas 14 proteins were uniquely secreted during the LL phase only, and 2 were uniquely secreted during the ML phase (Figure 2C). Of the 33 proteins identified, 27 proteins were shown to carry a signal peptide (SP) sequence, whereas six proteins lacked a SP; three of these SP-lacking proteins were predicted as non-classically secreted proteins, while three others were not predictable as secreted proteins (Table S4). Thirty-one of the 33 proteins also overlapped with proteins identified with the shotgun proteomic method and in silico prediction method as detailed in Section 3.6 below.

**Figure 3:**
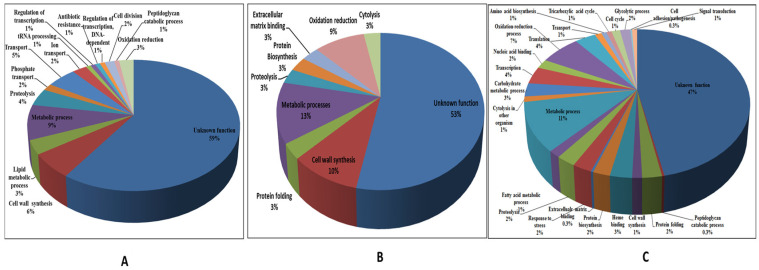
Functional distribution of the identified secretome proteins of *M. abscessus*. (Panel **A**) The proteins identified in silico. (Panel **B**) Proteins identified with 2D-GE. (Panel **C**) Proteins identified with the in-solution-digested shotgun proteomic method.

Functional distribution analysis of the proteins identified in the 2D-GE analysis (Figure 3B) revealed the following percent distribution profile across different functional categories: hypothetical (53%), metabolic processes (13%), cell wall synthesis (10%), proteolysis (3%), cytolysis (3%), protein synthesis (3%), oxidation–reduction processes (9%), protein folding (3%), and extracellular matrix binding (3%). Functional distribution analysis of the in silico-identified proteins and those identified with the bottom-up semi-quantitative proteomic method also confirmed the predominance of hypothetical proteins, followed by metabolic process proteins (Figure 3A,C).

### 3.3. Genome-Wide Identification of Secretory Proteins through the Generation and Screening of the M. abscessus Genomic phoA Fusion Library in M. smegmatis

In this study, we generated an *M. abscessus* genomic library in *E. coli* based on the insertion of size-selected genomic fragments (400–1500 bp), which were represented in 10,000 genomic clones. The transformation of a pool of recombinant plasmids from these clones into *M. smegmatis* mc2 155 yielded phoA-expressing clones that showed blue-colored colonies on the MB7H10-BCIP plates due to the activity of secreted alkaline phosphatase. Based on the intensity of the blue color, 23 blue colonies were chosen from the BCIP plates for further characterization.

Recombinant reporter plasmids that were isolated from the positive *M. smegmatis* clones and transformed into *E. coli* DH5α for amplification were verified for inserts through restriction analysis and sequenced using specific primers. BLAST analysis of the sequences against the *M. abscessus* genome yielded 23 specific ORFs (Table 5). The in vitro PhoA activity profile on the corresponding isolated 23 reporter clones—measured using p-nitrophenyl phosphate [pNPP] as a substrate—is presented in Table 5. The activity varied from 1.5 U to 12.0 U.

### 3.4. Genome-Wide Secreted Proteins of M. abscessus Predicted In Silico

A combination of bioinformatic tools were used for the prediction of the secretory signal sequence, as described in the Section 2, and a total of 116 secretory proteins were identified. The functional characterization of the identified secretory proteins based on the available annotation database for mycobacterial/bacterial proteins and a general protein database (www.ncbi.nlm.nih.gov (accessed on 17 January 2024)) is presented in Supplementary Table S1. N-terminal signal sequences and probable cleavage sites in the predicted proteins determined using SignalP analysis are also presented (Table S5). The signal peptide sequence was found to vary in length from 17 aa to 45 aa. Of the 116 secretion signal sequences, 90 contained a cleavage site with alanine at the −1 position. Most frequent motifs at the cleavage sites were found to contain the following amino acids: A-A (26%), A-E (9.48%), and A-D (18%). The remaining cleavage site motifs started with leucine (L), glycine (G), and proline (P).

### 3.5. Identification of Secretory Proteins Lacking a Signal Sequence

Based on the PubMed literature search for secretory proteins that lacked signal sequences, we identified five mycobacterial proteins, including early secretory antigenic target protein-6 (ESAT-6), superoxide dismutase (SOD), glutamine synthetase type I (GlnA1), and catalase/peroxidase (KatG). The search in the database for homologs of these proteins from tuberculous mycobacteria yielded nine candidates (Table 6) for sequence-lacking secreted proteins. Of these, two proteins, ESAT6 and CFP10, are known to utilize a special secretion system in tuberculous mycobacteria [25]. The mechanism(s) of secretion of the other signal-peptide-lacking mycobacterial proteins (Sod M, Gln A1, and KatG) is (are) not yet known.

### 3.6. Comparative Distribution of the Identified Secretome across the Different Identification Approaches Employed

**Figure 4:**
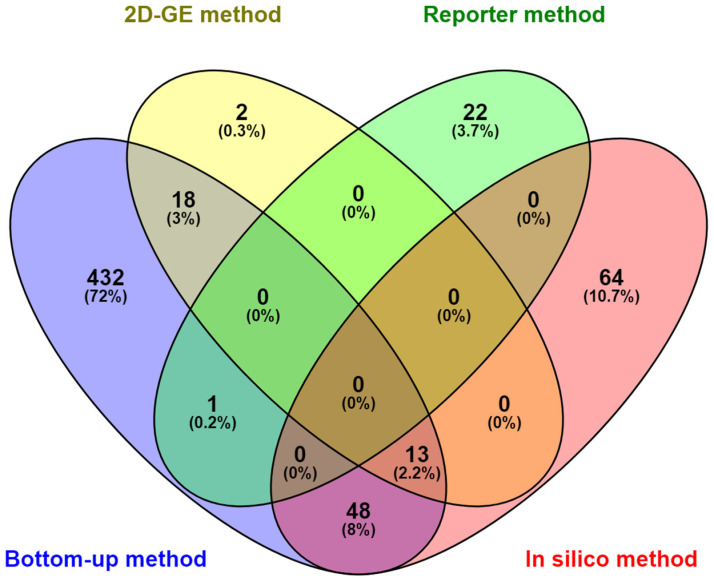
Venn diagram showing the differential distribution of the secretome of *M. abscessus* across different methods of identification. Secretome identification was conducted with four different approaches: the genomic reporter gene fusion library screening method, in silico analysis, 2D-GE proteomic analysis, and bottom-up shotgun proteomic method.

The secretome fractions identified with the four different methods are depicted in a comparative Venn diagram (Figure 4). A total of 698 proteins were identified with the approaches employed. The greatest number of proteins were identified with the bottom-up shotgun proteomic method (517 proteins), followed by the other methods in the following order: in silico method (125 proteins) > 2D-GE method (33 proteins) > reporter method (23 proteins). Of the 698 proteins, 432 proteins (72%) were exclusively identified with the bottom-up shotgun proteomic method, 2 proteins (0.3%) were exclusively identified with the 2D-GE proteomic method, 22 proteins (3.7%) were exclusively identified with the reporter method, and 64 proteins (10.7%) were exclusively identified with the in silico method. There were 13 common elements in the bottom-up method, 2D-GE method, and in silico method. Of the 33 proteins detected with 2D-GE proteomics, there was an overlap of 18 common elements between the bottom-up method and 2D-GE method. Of the 125 proteins detected with the in silico method, 48 common elements were found to overlap with the bottom-up method. Interestingly, out of the 23 proteins identified with the reporter method, only one protein overlapped with the bottom-up method. Notably, there were only 80 overlapping proteins across the different methods of identification, emphasizing the need for and significance of utilizing a multipronged approach to comprehensively capture the repertoire of secreted proteins.

The 125 computationally predicted secretory proteins included 116 with a secretion signal sequence and 9 without such a signal. In contrast, the majority of the 23 secreted proteins identified with the reporter gene fusion strategy were secreted through an unknown mechanism, as only two clones showed a known secretion mechanism, with one showing a typical signal peptide and the other showing a non-classical secretion score (Table 5).

## 4. Discussion

The putative assignment of biological roles to the identified individual proteins, which was separately depicted for the proteins predicted in silico (Figure 3A) and the proteomically identified proteins (Figure 3B,C), showed that the majority of the secretome had no known functional homologs in the database. These proteins that showed no similarity with the known functional proteins in the database were designated as ‘hypothetical’ proteins or ‘orphan’ proteins in this study. More than half (59%) of the secretory proteins identified via in silico prediction were found to be hypothetical. The experimental analysis of the culture filtrate proteins with 2D-GE and shotgun proteomics also revealed that over half of the secreted proteins had unknown functions (Figure 3B,C). This is an unusual observation that differs from reports on the majority of bacterial pathogens, including tuberculous species of mycobacteria [18,19].

Both the in silico and genetic approaches led to the identification of several secretory proteins with possible roles in cell wall biogenesis and virulence, such as the antigen 85 complex and penicillin-binding proteins (PBPs). These enzymes are known to induce both humoral and T-cell immune responses in patients infected with *M. tuberculosis*. Penicillin-binding proteins (PBPs) are membrane-bound proteins that are known to be involved in bacterial cell wall peptidoglycan synthesis [26].

Lipases such as phospholipase C and fatty acid-CoA racemase (FAR) were detected as secretory proteins in *M. abscessus* in this study (Figure 3, Table S1). During their infective stage, mycobacteria hydrolyze lipids, causing infection. For the degradation of extracellular and internal lipids, several lipase enzymes are produced [27,28].

Several identified secretory proteins of *M. abscessus* represented those involved in transport functions (Figure 3). Notably, ABC transporters, which facilitate the transport of a wide range of substrates, including ions, sugars, proteins, lipids, sterols, and drugs, were more abundant in *M. abscessus* than in *M. tuberculosis* (85 and 55, respectively). For instance, the transport of phosphate is mediated by Pst, a high-affinity ABC transport system. These systems are common to both pathogenic and nonpathogenic species of mycobacteria. Pst1 and Pst2 have been shown to be involved in the virulence of *M. tuberculosis* [29,30]. Surface-exposed phosphate transport receptor PstS-3 is a potent vaccine candidate and is being used as a DNA vaccine with Ag85A against *M. tuberculosis* infection [31].

Several secreted proteins identified in *M. abscessus* in this study, such as cutinase-like proteins, alkaline phosphatase, and catalase-peroxidase (KatG), are known to be involved in metabolic processes in *M. tuberculosis* and/or other microbial species (Figure 3). In *M. tuberculosis,* KatG is responsible for the activation of isoniazid (INH), a prodrug used as a front-line treatment for TB infection. KatG gene mutations are associated with other mutations in fabG1-inhA and oxyR-ahpC, conferring resistance/sensitivity against INH in *M. tuberculosis* strains [32,33]. Phosphoribosyl-ATP pyrophosphohydrolase (hisE encoded), the second enzyme of the histidine biosynthesis pathway found in many microorganisms and plants, has been recognized as a potential drug target in *M. tuberculosis* [34]. Glutamine synthetase (GlnA1) is an important drug target and a major secreted protein of *M. tuberculosis*. As no leader sequence was found in this protein, it may be an autotransporter [35]. GlnA1 plays a key role in nitrogen assimilation by incorporating NH3 into glutamate to make glutamine [36,37]. Mutations in this gene in *M. tuberculosis* attenuated intracellular growth both in human macrophage cell lines and in a guinea pig model of pulmonary TB [11,38]. GlnA1 is also involved in the synthesis of PLG, an important cell wall constituent that is specific to pathogenic bacteria [11,37].

Some of the secreted proteins identified in *M. abscessus* corresponded to those known to have a role in antibiotic resistance in mycobacteria (Figure 3). These organisms secrete detectable amounts of beta-lactamase in culture media, and this acts as penicillinase [39]. The low permeability of beta-lactam antibiotics, the low affinity of these antibiotics to penicillin-binding proteins, and the secretion of beta-lactamase in culture media are collectively responsible for the resistance of mycobacteria to beta-lactam antibiotics. *M. abscessus* has nine probable beta-lactamase-like proteins, as compared to *M. tuberculosis,* which has three beta-lactamases [40]. This higher number of beta-lactamase genes may be responsible for the conferral of greater drug resistance to *M. abscessus*.

The 2D-GE analysis revealed a secretory protein, D-alanyl-D-alanine dipeptidase, in *M. abscessus* (Figure 3 and Table 4), which, due to its protease activity, is known to confer vancomycin resistance in enterococci and has also been reported in *Salmonella enterica* [41]. Proteases are important virulence factors found in the majority of pathogenic microorganisms and are known to be predominant in several pathogenic *Mycobacterium* species.

The PE protein was identified with the reporter gene fusion method, though it evaded in silico prediction. This protein belongs to the multigene protein families of PPE, PE, and PE-PGRS. These proteins occupy 10% of the *M. tuberculosis* genome, and their homologs are found in almost all mycobacterial species. The PE protein is a unique protein for mycobacteria. In the *M. abscessus* genome, there are six proteins from the PE and PPE families each. These proteins are responsible for the antigenic variation in mycobacterium that may help in pathogenesis and adaptation to host cells [42]. A strong antibody response was found against the PE-PGRS62 protein in active and latent tuberculosis [43]. PE proteins have been shown to be localized in the cell wall of mycobacteria [44]. Recently, it was demonstrated that PE proteins function as triacylglycerol (TAG) hydrolase. Due to the surface exposure of the PE protein, it could be a potential vaccine candidate. Further, recent studies showed that PE/PPE proteins in mycobacteria are involved in nutrient transport [45]. A recent study showed that the M. tb PE6 protein (Rv0335c), a secretory protein effector through TLR4 interaction, induced the secretion of proinflammatory cytokines TNF-α, IL-12, and IL-6 through the activation of the canonical NFĸB signaling pathway. In addition, it induced apoptosis via Bax, cytochrome C, and pcMyc production [46].

Several immunogenic proteins are secreted into extracellular culture environments. These secreted proteins provide strong cell-mediated and delayed-type hypersensitivity (DTH) reactions. MPT64 is a 23 kDa secreted protein found in the culture filtrates of *M. tuberculosis* that causes both cell-mediated immune response and a DTH response [47]. In a recent study, MPT64 impaired the ER-mediated unfolded response in macrophages [48]. This protein was used in a trial of vaccination against *M. tuberculosis* infection. *M. abscessus* has two MPT 64-like secreted proteins, mpt64 and mpt53. They can be investigated as vaccine candidates for *M. abscessus* infections.

In summary, the current study generated a knowledge base concerning the putative secreted protein effectors of *M. abscessus* based on their functional homologs in other bacterial pathogens—particularly *M. tuberculosis*. However, the large functionally “orphan” fraction of the secretome revealed in this study is a valuable revelation regarding the putative novel secreted effectors in *M. abscessus* awaiting future research.

## 5. Conclusions

The comprehensive identification of the secreted proteins of *M. abscessus* in this study is a significant step forward in the ongoing efforts to understand the virulence and drug resistance mechanisms and the antigen repertoire for the development of new vaccines and therapeutics for this emerging lung pathogen with unusual antibiotic resistance. The results of this study emphasize the importance of employing a combination of both in silico prediction techniques and laboratory-based strategies (in conjunction with searches of the literature and databases) for a comprehensive revelation of the secretome of this mycobacterial species. While over half of the secreted proteins identified in this study were ‘hypothetical’ or ‘orphan’ (those with yet unknown functions), the remaining candidates belonged to different functional categories that coincided with roles in virulence, immunity, and antibiotic resistance. Future research on the identified proteins may focus on the deorphanization of the hypothetical proteins and experimental characterization of the predicted functions of individual proteins using gene deletion or overexpression strategies with in vitro and in vivo models of infection or immunity. Specific efforts toward the identification of novel B-cell and T-cell antigens in the unveiled secretome will help in the development of rapid diagnostics and vaccine candidates for infections of this emerging pathogen.

## Figures and Tables

**Table 1:** Overview of the total secreted proteins identified using bottom-up shotgun proteomics.

Category	Proteins Identified	“Tab” *	Array **
**Proteins identified in all phases (5 min, EL, ML, LL)** (>2-fold and *p* < 0.05 compared to the 5 min sample)	517	overview_spectrum_counts	E4–F520
**Proteins identified in EL** (>2-fold and *p* < 0.05 compared to the 5 min sample)	107	Figure 1 (Panel B and C)	B6–C112
**Proteins identified in ML** (>2-fold and *p* < 0.05 compared to the 5 min sample)	201	Figure 1 (Panel B and C)	G6–H206
**Proteins identified in LL** (>2-fold and *p* < 0.05 compared to the 5 min sample)	163	Figure 1 (Panel B and C)	L6–M168
**Proteins identified ONLY in EL**	0	Table S6	N.A.
**Proteins identified ONLY in ML**	41	Table S6	B162–C202
**Proteins identified ONLY in LL**	8	Table S6	B203–C210
**Overlap**			
**EL vs. ML** *(EL/5 min; >2-fold and p < 0.05)* vs. *(ML/5 min; >2-fold and p < 0.05)*	6	Table S6	B105–C110
**ML vs. LL** *(ML/5 min; >2-fold and p < 0.05)* vs. *(LL/5 min; >2-fold and p < 0.05)*	51	Table S6	B111–C161
**EL vs. LL** *(EL/5 min; >2-fold and p < 0.05)* vs. *(LL/5 min; >2-fold and p < 0.05)*	0	Table S6	N.A.
**EL vs. ML vs. LL** *(EL/5 min; >2-fold and p < 0.05)* vs. *(ML/5 min; >2-fold and p < 0.05) vs.* *(LL/5 min; >2-fold and p < 0.05)*	99	Table S6	B6–C104

* “Tab” refers to the spreadsheet tabs in Supplementary Excel File Table S6; ** Array refers to the area occupied in the above-mentioned Excel spreadsheet. N.A. refers to not applicable.

**Table 2:** Differential secretory proteomes of different growth phases.

Category	Proteins Identified	Number of Differentially Secreted Proteins (DSPs) (*p* < 0.05)	Abundance of Differentially Secreted Proteins (DSPs) (*p* < 0.05)	
			Fold Change (FC)	
			>2	<2
EL/5 min	162	107	107 (105) *	0
ML/5 min	324	201	201 (197)	0
LL/5 min	465	163	163 (158)	0
ML/EL	329	180	142 (140)	0
LL/EL	470	140	118 (115)	0
LL/ML	508	33	9 (8)	6

* Numbers in parentheses represent the numbers of proteins identified after protein isoform grouping with Scaffold. Data were compiled based on the Supplementary Materials in Tables S6 and S7.

**Table 3:** Comparison of the differentially secreted proteins (DSPs) (FC > 2; *p* < 0.05) identified in different growth phases using bottom-up proteomics.

Specific Growth Phases Compared and Comparisons between Growth Phases	Number of Common DSPs Overlapping between Growth Phases	Number of DSPs Unique to a Specific Growth Phase or Combination of Growth Phases
[EL/5 min] vs. [ML/5 min] vs. [LL/5 min]		
[EL/5 min]	N.A.	0
[ML/5 min]	N.A.	41
[LL/5 min]	N.A.	8
[EL/5 min] and [ML/5 min]	105	6
[ML/5 min] and [LL/5 min]	150	51
[EL/5 min] and [LL/5 min]	0	0
[EL/5 min], [ML/5 min], and [LL/5 min]	99	N.A.
[EL/5 min] vs. [ML/EL] vs. [LL/EL]		
[EL/5 min]	N.A.	33
[ML/EL]	N.A.	43
[LL/EL]	N.A.	8
[EL/5 min] and [ML/EL]	54	8
[EL/5 min] and [LL/EL]	64	18
[ML/EL] and [LL/EL]	89	43
[EL/5 min], [ML/EL], and [LL/EL]	46	N.A.

Numbers represent proteins identified after protein isoform grouping with Scaffold and were compiled based on the Supplementary Tables. A detailed listing of the identified DSPs is given in Supplementary Tables S6 and S7. Abbreviations: EL (culture in the early-log growth phase), ML (culture in the mid-log growth phase), LL (culture in the late-log growth phase), 5 min (culture at 5 min post-inoculation). The forward slash represents a comparison between two phases. N.A. refers to not applicable.

**Table 4:** Identification of secretory proteins of *M. abscessus* with the 2D-GE approach.

Spot ID ^a^	Protein ID	Protein Name	Locus Name	MW/PI	Peptides	F ^b^	Culture Phase ^c^
ML4, LL4, LL5	B1MCR7 *	Uncharacterized protein	MAB_2960	73.5/5.03	5, 2, 2	3	ML, LL
ML5, ML6, ML7, LL6, LL7, LL8, LL9, LL11	B1MIY6 *	Uncharacterized protein	MAB_4284c	62.9/5.01	3, 4, 4, 3, 2, 4, 3, 4	8	ML, LL
ML10, LL13, LL14	B1MMK7 *	Uncharacterized protein	MAB_4924	32/5.02	4, 5, 5	3	ML,LL
ML13, ML14, ML16, LL17, LL18, LL19	B1MEL2 *	Antigen 85-A	MAB_0176	35.8/6.57	3, 5, 4, 3, 5, 6	6	ML, LL
ML13, ML14, LL17, LL19, LL20,	B1MEL1 *	Antigen 85-C	MAB_0175	34.7/6.8	5, 3, 4, 5, 3	5	ML, LL
ML13, ML14, ML16, ML18, ML19	B1MEL3 *	Antigen 85-A//B/C	MAB_0177	34.9/6.13	2, 3, 3, 3, 3	5	ML, LL
ML15, LL21	B1MNL7 *	Immunogenic protein MPT64	MAB_1835c	29.4/6.19	4, 6	2	ML, LL
ML17, LL22	B1MDW2 *	Putative secreted hydrolase	MAB_3355	28.9/6.3	2, 4	2	ML,LL
ML18, ML19, LL23, LL24	B1MMY7 *	Uncharacterized protein	MAB_1614	22.3/4.88	4, 2, 3, 3	4	ML, LL
ML20, LL29, LL30	B1MFV4 *	Uncharacterized protein	MAB_0405c	20.2/5.99	3, 2, 3	3	ML, LL
ML21, LL30	B1MBL9 *	Peptidyl-prolyl cis-trans isomerase	MAB_2559c	19.1/5.06	2, 2	2	ML,LL
ML22, LL36	B1MCH9 *	Uncharacterized protein	MAB_2871c	14.8/5.12	4, 4	2	ML, LL
ML23	B1MIR3 *	Uncharacterized protein	MAB_4211c	14/5.14	2	1	ML
ML25, ML27, LL25, LL26, LL27	B1MBM0 *	Uncharacterized protein	MAB_2560	20.5/5.88	4, 3, 4, 2, 6	5	ML, LL
ML27, LL42	B1MNM5 *	D-alanyl-D-alanine dipeptidase	MAB_1843	23.8/6.92	3, 6	2	ML, LL
ML28	B1MFV1 *	Uncharacterized protein	MAB_0402	25.2/6.71	3	1	ML
LL3, LL5	B1MJB0 *	Uncharacterized protein	MAB_0974	61.2/5.39	3, 5	1	LL
LL12	B1MB96 *	Uncharacterized protein	MAB_2436	34.8/6.06	4	1	LL
LL12	B1MJN7	Aldehyde dehydrogenase family protein	MAB_4322	47.2/4.82	2	1	LL
ML11, LL16	B1MKW1 *	Uncharacterized protein	MAB_4537c	35.7/5.88	2, 3	2	ML,LL
ML13, LL17	B1MDF1	Elongation factor Ts	MAB_3195c	29.1/5.08	2, 4	2	ML,LL
ML13, LL18, LL19	B1MDV5 *	Uncharacterized protein	MAB_3348	29.5/6.34	2, 2, 2	3	ML,LL
LL19	B1MEC9 *	Uncharacterized protein	MAB_0093	28.4/5.34	2	2	LL
LL25	B1MG92 *	Cutinase	MAB_3763	23.5/6.3	2	1	LL
LL28	B1MBD4 *	Uncharacterized protein	MAB_2474	19.1/8.41	3	1	LL
LL29	B1MDB0	Uncharacterized protein	MAB_3154c	21.7/5.06	2	1	LL
LL31, LL32	B1MGD1 *	Uncharacterized protein	MAB_3801c	18.5/6	3, 2	2	LL
LL38	B1MDK0 *	Soluble secreted antigen MPT53	MAB_3243	19.1/9.57	2	1	LL
LL39	B1MAH1 *	Hypothetical low molecular weight antigen Mtb12	MAB_2161c	17.3/4.81	3	1	LL
LL39	B1MDK6 *	Uncharacterized protein	MAB_3249	16.2/6.58	2	1	LL
LL39	B1MIH2	Probable aldo/keto reductase	MAB_4120c	35.3/5.11	2	1	LL
LL45	B1MCS9 *	Uncharacterized protein	MAB_2972	17.6/7.06	3	1	LL
LL47	B1MMY9 *	Uncharacterized protein	MAB_1616	20.3/7.97	3	1	LL

^a^ Spots that are indicated in Figure 2 but did not yield positive identification according to LC/MS are not listed. ^b^ Frequency describes the number of times that the same protein was detected (as variants) at different spots. ^c^ Cultures grown to mid-log phase (ML) and late-log phase (LL). * Proteins that were also identified with the shotgun proteomic method.

**Table 5:** Identification of secretory proteins based on the *pho*A reporter gene fusion library screening approach.

Clone ID	Gene ID	Corresponding Protein ID	Putative Function	Alkaline Phosphatase Activity
1	MAB_3995	B1MHI8	Hypothetical protein	3.53 ± 0.50
2	MAB_1483	B1MLZ2	Ribonuclease PH	4.00 ± 1.00
4	MAB_0845	B1MIB7	Fatty-acid-CoA racemase Far	3.50 ± 0.50
5	MAB_3868c	B1MH61	DNA-directed RNA polymerase beta’ chain	5.86 ± 1.20
6	MAB_1496c	B1MM05	TetR family transcriptional regulator	7.56 ± 0.75
7	MAB_1902	B1MNT4	Hypothetical protein	12.00 ± 1.00
8	MAB_4022c	B1MHL5	Oxidoreductase	8.60 ± 1.20
11	MAB_1387	B1MLP6	Putative esterase/lipase/beta-lactamase	1.50 ± 0.50
12	MAB_3282c	B1MDN9	Lipase	1.90 ± 0.10
13	MAB_3233	B1MDJ0	D-alanyl-D-alanine carboxypeptidase DacB	6.90 ± 1.01
14	MAB_4001c	B1MHJ4	Putative 2-nitropropane dioxygenase, NPD	3.36 ± 0.60
16	MAB_0351	B1MFQ0	Catalase	5.86 ± 0.23
18 *	MAB_0087c	B1MEC3	Alkaline phosphatase	6.26 ± 0.60
20	MAB_4017c	B1MHL0	Probable monooxygenase	4.80 ± 1.50
20a	MAB_1064	B1MJJ8	Bifunctional phospho ribosylaminoimidazole carboxamide formyltransferase/IMP cyclohydrolase	4.16 ± 1.04
21	MAB_4011c	B1MHK4	Hypothetical protein	3.43 ± 0.50
21a	MAB_3366	B1MDX3	Possible glycosyl transferase	6.16 ± 0.70
21b	MAB_2131	B1MPG1	Phosphoribosyl-ATP pyrophosphatase	4.50 ± 0.50
22 ^ψ^	MAB_1999	B1MP30	Hypothetical protein	3.50 ± 0.50
26	MAB_3907c	B1MHA0	LuxR transcriptional regulator	3.83 ± 0.20
27	MAB_4384	B1MJU8	Putative transcriptional regulator, TetR family	5.23 ± 0.68
28	MAB_1295c	B1MLF6	O-methyltransferase OMT	3.50 ± 0.50
29	MAB_1589	B1MMW2	TetR family transcriptional regulator	6.16 ± 1.04

The ± value indicates the standard deviation of the means of triplicate experiments. * This clone contained a typical signal peptide sequence. **^ψ^** This clone gave a non-classical secretion score.

**Table 6:** Secretory proteins lacking signal sequences that were identified based on homologs in *M. tuberculosis*.

Serial Number	Name of Protein	Locus Name	Function
1	ESAT-6-like protein (10 kDa antigen	MAB_0666	Immunogenic protein
2	ESAT-6-like protein esxH	MAB_2228c	Immunogenic protein
3	ESAT-6-like protein	MAB_0049	Immunogenic protein
4	Superoxide dismutase SodM	MAB_3957	Catalyzes free radicals (O_2_^−^) to hydrogen peroxide and molecular oxygen
5	Superoxide dismutase	MAB_4184c	Catalyzes free radicals (O_2_^−^) to hydrogen peroxide and molecular oxygen
6	Superoxide dismutase (Mn)	MAB_0118c	Catalyzes free radicals (O_2_^−^) to hydrogen peroxide and molecular oxygen
7	Glutamine synthetase, type I (GlnA1)	MAB_1933c	Catalyzes the formation of glutamine in an ATP-dependent assimilation of ammonia into glutamate
8	Catalase/peroxidase (KatG)	MAB_2470c	Peroxidase activity
9	CFP29, the 29 kDa antigen (bacteriocin CFP29)	MAB_0699c	Immunogenic protein

## Data Availability

The data supporting the reported results can be found in the included Supplementary Files.

## Supplementary Materials

The following supporting information can be downloaded at: https://www.mdpi.com/article/10.3390/microorganisms12020378/s1, Supplementary Word File (Tables S1–S5) and Supplementary Excel File (Tables S6 and S7; sorted data for Figure 1B–D and Figure 4): overview of spectral counts.
