# Supplementary material for: Predominantly Orphan Secretome in the Lung Pathogen Mycobacterium abscessus Revealed by a Multipronged Growth-Phase-Driven Strategy
Source: Microorganisms. 2024 Feb 12;12(2):378. doi: 10.3390/microorganisms12020378 (PMC10892769; doi:10.3390/microorganisms12020378)
Supplement: Supplementary file 1 [file microorganisms-12-00378-s001.zip › microorganisms-2855921-supplementary.pdf]

Predominantly Orphan Secretome in the Lung Pathogen Mycobacterium abscessus Revealed by a Multipronged Growth Phase-Driven Strategy

Harish Chandra[1], Manish K. Gupta[1], Ying-Wai Lam[2] and Jagjit S. Yadav [1,*]

[1] Pulmonary/Microbial Pathogenesis Laboratory, Department of Environmental and Public Health Sciences, University of Cincinnati College of Medicine, Cincinnati, OH 45267, USA; chandrhh@ucmail.uc.edu (H.C.)
[2] Vermont Biomedical Research Network Proteomics Facility, University of Vermont, Burlington, VT 05405, USA
* Correspondence: jagjit.yadav@uc.edu; Tel.: +1-513-558-4806

SUPPLEMENTARY INFORMATION

Table S1: Annotated secreted proteins of *M. abscessus* predicted in silico.

Serial #	Protein ID	Protein name	Locus name	Gene symbol	Molecular function	Biological Function
1	B1MEL2	Antigen 85-A precursor	MAB_0176	fbpA	Mycolyltransferase	Cell wall biogenesis
2	B1MEL3	Antigen 85-A/B/C precursor	MAB_0177	fbpA	Mycolyltransferase	Cell wall biogenesis
3	B1MMV2	Antigen 85-B precursor	MAB_1579	fbpA	Mycolyltransferase	Cell wall biogenesis
4	B1MEL1	Antigen 85-C precursor	MAB_0175	-	Mycolyltransferase	Cell wall biogenesis
5	B1MFV7	Probable bifunctional membrane-associated penicillin-binding protein PonA2/glycosyl transferase	MAB_0408c	ponA2	Glycosyltransferase Hydrolase Transferase	Cell wall biogenesis /degradation
6	B1MP31	Probable penicillin-binding membrane protein PbpB	MAB_2000	pbpB	Hydrolase	Antibiotic resistance/cell wall biogenesis/degradation
7	B1MEK4	Putative N-acetymuramoyl-L-alanine amidase	MAB_0168c	-	Protein binding	Peptidoglycan catabolic process
8	B1MKI9	Conserved hypothetical protein (lipolytic enzyme G-D-S-L?)	MAB_1193c	-	Hydrolase	Lipid metabolic process
9	B1MGX0	Probable non-hemolytic phospholipase C	MAB_0555	-	Hydrolase	Lipid catabolic process
10	B1MMD5	Phosphate ABC transporter, periplasmic protein	MAB_4852c	pstS3	Inorganic phosphate transmembrane transporter activity	Phosphate transport Stress response Transport
11	B1MDZ7	Probable FeIII-dicitrate-binding periplasmic lipoprotein	MAB_3390	fecB	Iron ion transmembrane transporter activity	High-affinity iron ion transport
12	B1MHE3	Probable low-affinity inorganic phosphate transporter	MAB_3950c	pitA	Transporter activity	Phosphate transport

13	B1MC95	Probable molybdenum ABC transporter, periplasmic	MAB_2787c	-	Molybdate transmembrane-transporting ATPase activity	Molybdate ion transport
14	B1MLN1	Probable sugar ABC transporter, sugar-binding protein LpqY	MAB_1372	lpqY	Transporter activity	Transport
15	B1MGZ1	Putative ABC transporter solute binding protein	MAB_0577c	-	Transporter activity	Transport
16	B1MBC2	Putative sulfonate ABC transporter, periplasmic protein	MAB_2462	-	Transporter activity	Sulfur metabolic process
17	B1MGZ0	Putative ABC transporter, ATP-binding protein	MAB_0576c		ATPase activity	Transport
18	B1MIT8	Putative amino acid ABC transporter, substrate-binding protein	MAB_4236c	-	Transporter activity	Transport
19	B1MD08	Putative glutamate ABC transporter, periplasmic protein	MAB_3051	-	Transporter activity	Transport
20	B1MJV4	Putative ABC transporter, periplasmic substrate-binding	MAB_4390	-	ATPase activity	Transport
21	B1MH02	Porphyromonas-type peptidyl-arginine deiminase	MAB_0588c	-	Hydrolase	Nitrogen compound metabolic process
22	B1MBK6	Probable amidohydrolase	MAB_2546	-	Hydrolysis	Metabolism
23	B1MG92	Probable cutinase cut2 precursor	MAB_3763	cut2	Hydrolase Serine esterase	Metabolic process
24	B1MG94	Probable cutinase cut3 precursor	MAB_3765	cut3	Cutinase activity	Metabolic process
25	B1MG95	Probable cutinase cut3	MaB_3766	Cut3	Hydrolase activity	Metabolic process
26	B1MG78	Putative phosphodiesterase/alkaline phosphatase	MAB_3749	-	Phosphatase activity	Metabolic process
27	B1MCI3	Beta-lactamase precursor (Penicillinase)	MAB_2875	blaC	Hydrolase	Antibiotic resistance
28	B1MBQ0	Putative polysaccharide deacetylase	MAB_2590	-	Hydrolase	Carbohydrate metabolic process
29	B1MDW2	Putative secreted hydrolase	MAB_3355	-	Hydrolase	Carbohydrate metabolic process
30	B1MH82	Putative triacylglycerol lipase precursor	MAB_3889	-	Hydrolase	Lipid degradation
31	B1MHZ7	Putative triacylglycerol lipase precursor	MAB_0723c	-	Hydrolase	Lipid degradation
32	B1MK31	Sulfatase family protein	MAB_4469c	-	Sulfuric ester hydrolase activity	Metabolic process
33	B1MKF7	Conserved hypothetical protein	MAB_1161c	-	Peroxidase activity	Hypothetical

34	B1MNM5	Probable D-alanyl-D-alanine dipeptidase	MAB_1843	lpqR	Dipeptidase activity	Proteolysis
35	B1MIR4	Probable lipoprotein aminopeptidase LpqL	MAB_4212c	lpqL	Peptidase activity	Proteolysis
36	B1MFX3	Putative protease	MAB_0424	-	Protease	Peptidase activity
37	B1MHA3	Serine peptidase	MAB_3910	-	Protease	Proteolysis
38	B1MKX8	Putative metallopeptidase	MAB_4554c	-	Metalloprotease	Proteolysis
39	B1MG10	Probable penicillin-binding protein DacB1	MAB_3681	dacB1	Carboxypeptidase	Serine-type D-Ala-D-Ala carboxypeptidase activity
40	B1MJH6	Probable cytochrome c oxidase polypeptide I	MAB_1042c	ctaD	Oxidoreductase	Electron transport
41	B1MKR7	Probable oxidase (copper-binding protein)	MAB_1271c	-	Oxidoreductase	Oxidation reduction
42	B1MGM8	Probable oxidase (copper-binding protein)	MAB_0463c	-	Oxidoreductase	Oxidation reduction
43	B1MFR7	Short-chain dehydrogenase/reductase	MAB_0368	-	Oxidoreductase	Oxidation reduction
44	B1MAP1	Probable PE family protein	MAB_0046	PE5	Function not known	Antigenic
45	B1MC08	Probable pseudouridine synthase RluD	MAB_2699c	-	Isomerase	Pseudouridine synthesis
46	B1MJ07	Probable resuscitation-promoting factor RpfA	MAB_0869c	rpfA	Signal transduction	For growth
47	B1MC58	Probable transcriptional regulatory protein	MAB_2750c	-	Transcription factor activity	Regulation of transcription, DNA-dependent
48	B1MKG0	Putative conserved lipoprotein LpqU	MAB_1164	lpqU	Function not known	Hypothetical protein
49	B1MEK5	Putative exported repetitive protein precursor	MAB_0169c	pirG	Multiplication	Intracellular growth
50	B1MP05	Putative secreted protein	MAB_1974	-	Function not known	Hypothetical protein
51	B1MGT6	Putative tRNA(Ile)-lysidine synthase	MAB_0521	mesJ	Ligase	tRNA processing
52	B1MIN7	Putative tuberculin related peptide	MAB_4185c	-	Function not known	Hypothetical protein
	**Hypothetical proteins with Unknown functions**: MAB_4782, MAB_0743c, MAB_0734, MAB_4576c, MAB_0684c, MAB_0620c, MAB_1553c, MAB_1543, MAB_1616, MAB_4281c, MAB_4280c, MAB_4924, MAB_4915c, MAB_4914c, MAB_4903, MAB_3031c, MAB_0565c, MAB_0560, MAB_4211c, MAB_4192, MAB_4037, MAB_2697c, MAB_1323, MAB_1322, MAB_2878c, MAB_2871c, MAB_1511, MAB_2594, MAB_2805c, MAB_2800, MAB_2799, MAB_2798c, MAB_1261, MAB_0360, MAB_0353, MAB_4062c, MAB_4061c, MAB_2456, MAB_2453c, MAB_2420c, MAB_1130, MAB_4114, MAB_2786, MAB_2785, MAB_1363, MAB_2329c, MAB_1026c, MAB_1003c, MAB_3532, MAB_0957, MAB_3472, MAB_3454, MAB_3493, MAB_2113, MAB_3305c, MAB_3348, MAB_0884c, MAB_0848c, MAB_3264c, MAB_3215c, MAB_3165c, MAB_3154c, MAB_0093, MAB_0063c					

Table S2: Identification of secretory proteins in the mid-log-phase culture of *M. abscessus* with 2D-GE proteomics.

Spot ID #	Protein ID #	Protein Name	Locus Name	Score	Coverage	Proteins	Unique peptides	Peptides	PSMs	AAs	MW [KDa]	Calc. PI
**ML4**	B1MCR7	Uncharacterized protein	MAB_2960	14.67	9.53	1	5	5	5	682	73.5	5.03
**ML5**	B1MIY6	Uncharacterized protein	MAB_4284c	19.52	6.55	1	3	3	5	687	62.9	5.01
**ML6**	B1MIY6	Uncharacterized protein	MAB_4284c	25.59	9.17	1	4	4	7	687	62.8	5.008
**ML7**	B1MIY6	Uncharacterized protein	MAB_4284c	34.92	9.17	1	4	4	9	687	62.9	5.01
**ML10**	B1MMK7	Uncharacterized protein	MAB_4924	20.1	14.02	1	4	4	7	321	32	5.02
**ML11**	B1MKW1	Uncharacterized protein	MAB_4537c	4.38	4.5	1	2	2	2	333	35.7	5.88
**ML13**	B1MEL1	Antigen 85-C	MAB_0175	44.88	16.98	1	4	5	12	324	34.7	6.8
**ML13**	B1MEL2	Antigen 85-A	MAB_0176	17.79	10.21	1	2	3	6	333	35.8	6.57
**ML13**	B1MEL3	Antigen 85-A/B/C	MAB_0177	10.37	5.85	1	2	2	3	325	34.9	6.13
**ML13**	B1MDF1	Elongation factor Ts	MAB_3195c	10.77	8.66	1	2	2	3	277	29.1	5.08
**ML13**	B1MDV5	uncharacterized protein	MAB_3348	6.96	10.10	1	2	2	2	287	29.5	6.34
**ML14**	B1MEL2	Antigen 85-A	MAB_0176	36.52	16.22	1	3	5	12	333	35.8	6.57
**ML14**	B1MEL1	Antigen 85-C	MAB_0175	17.34	8.02	1	2	3	5	324	34.7	6.8
**ML14**	B1MEL3	Antigen 85-A//B/C	MAB_0177	14.31	12	1	2	3	4	325	34.9	6.13
**ML15**	B1MNL7	Immunogenic protein MPT64	MAB_1835c	15.96	14.55	1	4	4	6	275	29.4	6.19
**ML16**	B1MEL2	Antigen 85-A	MAB_0176	21.59	12.91	1	4	4	7	333	35.8	6.57
**ML16**	B1MEL3	Antigen 85-A//B/C	MAB_0177	8.41	8.62	1	3	3	3	325	34.9	6.13
**ML17**	B1MDW2	Putative secreted hydrolase	MAB_3355	14.2	12.59	1	2	2	4	286	28.9	6.3
**ML18**	B1MMY7	Uncharacterized protein	MAB_1614	29.25	19.25	1	4	4	8	213	22.3	4.88
**ML19**	B1MMY7	Uncharacterized protein	MAB_1614	14.6	15.96	1	2	2	3	213	22.3	4.88
**ML20**	B1MFV4	Uncharacterized protein	MAB_0405c	22.72	13.33	1	3	3	8	195	20.2	5.99
**ML21**	B1MBL9	Peptidyl-prolyl cis-trans isomerase	MAB_2559c	19	17.86	1	2	2	5	196	19.1	5.06
**ML22**	B1MCH9	Uncharacterized protein	MAB_2871c	48.17	22.86	1	4	4	15	140	14.8	5.12
**ML23**	B1MIR3	Uncharacterized protein	MAB_4211c	9.37	22.9	1	2	2	3	131	14	5.14
**ML25**	B1MBM0	Uncharacterized protein	MAB_2560	30.97	27.36	1	4	4	9	201	20.5	5.88

**ML27**	B1MNM5	D-alanyl-D-alanine dipeptidase	MAB_1843	17.51	19.37	1	3	3	5	222	23.8	6.92
**ML27**	B1MBM0	Uncharacterized protein	MAB_2560	17.1	21.89	1	3	3	5	201	20.5	5.88
**ML28**	B1MFV1	Uncharacterized protein	MAB_0402	18.92	16.24	1	3	3	6	234	25.2	6.71

Table S3: Identification of secretory proteins in the late-log-phase culture of *M. abscessus* with 2D-GE proteomics.

Spot ID #	Protein ID #	Protein Name	Locus Name	Score	Coverage	Proteins	Unique peptides	Peptides	PSMs	AAs	MW [KDa]	Calc. PI
**LL3**	B1MJB0	Uncharacterized protein	MAB_0974	9.39	8.09	1	3	3	3	606	61.2	5.39
**LL4**	B1MCR7	Uncharacterized protein	MAB_2960	8.2	2.49	1	2	2	3	682	73.5	5.03
**LL5**	B1MJB0	Uncharacterized protein	MAB_0974	12.93	7.1	1	3	3	4	606	61.2	5.39
**LL5**	B1MCR7	Uncharacterized protein	MAB_2960	8.12	2.49	1	2	2	4	682	73.5	5.03
**LL6**	B1MIY6	Uncharacterized protein	MAB_4284c	22.85	6.11	1	3	3	7	687	62.9	5.01
**LL7**	B1MIY6	Uncharacterized protein	MAB_4284c	15.3	3.49	1	2	2	4	687	62.9	5.01
**LL8**	B1MIY6	Uncharacterized protein	MAB_4284c	36.53	9.17	1	4	4	9	687	62.9	5.01
**LL9**	B1MIY6	Uncharacterized protein	MAB_4284c	15.73	7.57	1	3	3	4	687	62.9	5.01
**LL11**	B1MIY6	Uncharacterized protein	MAB_4284c	25.53	9.17	1	4	4	7	687	62.9	5.01
**LL12**	B1MB96	Uncharacterized protein	MAB_2436	24.95	11.63	1	4	4	8	344	34.8	6.06
**LL12**	B1MJN7	Aldehyde dehydrogenase family protein	MAB_4322	5.3	4.92	1	2	2	2	447	47.2	4.82
**LL13**	B1MMK7	Uncharacterized protein	MAB_4924	28.85	23.05	1	5	5	9	321	32	5.02
**LL14**	B1MMK7	Uncharacterized protein	MAB_4924	36.53	24.92	1	5	5	12	321	32	5.02
**LL16**	B1MKW1	Uncharacterized protein	MAB_4537c	18.73	13.81	1	3	3	6	333	35.7	5.88
**LL17**	B1MEL1	Antigen 85-C	MAB_0175	29.86	13.27	1	3	4	8	324	34.7	6.8
**LL17**	B1MDF1	Elongation factor Ts	tsf	22.48	18.05	1	4	4	6	277	29.1	5.08
**LL17**	B1MEL2	Antigen 85-A	MAB_0176	19.25	10.21	1	2	3	6	333	35.8	6.57
**LL18**	B1MEL2	Antigen 85-A	MAB_0176	41.42	16.22	1	4	5	14	333	35.8	6.57
**LL18**	B1MEL3	Antigen 85-A/B/C	MAB_0177	20.45	12	1	2	3	7	325	34.9	6.13

**LL18**	B1MDV5	Uncharacterized protein	MAB_3348	17.62	10.1	1	2	2	5	287	29.5	6.34
**LL19**	B1MEL2	Antigen 85-A	MAB_0176	62.34	18.92	1	4	6	19	333	35.8	6.57
**LL19**	B1MEL3	Antigen 85-A/B/C	MAB_0177	40.29	12	1	2	3	11	325	34.9	6.13
**LL19**	B1MEL1	Antigen 85-C	MAB_0175	32.15	16.98	1	4	5	9	324	34.7	6.8
**LL19**	B1MEC9	Uncharacterized protein	MAB_0093	13.39	8.85	1	2	2	4	260	28.4	5.34
**LL19**	B1MDV5	Uncharacterized protein	MAB_3348	11.23	10.1	1	2	2	3	287	29.5	6.34
**LL20**	B1MEL1	Antigen 85-C	MAB_0175	19.15	11.42	1	3	3	5	324	34.7	6.8
**LL21**	B1MNL7	Immunogenic protein MPT64	MAB_1835c	38.19	28.73	1	6	6	12	275	29.4	6.19
**LL22**	B1MDW2	Putative secreted hydrolase	MAB_3355	20.54	22.03	1	4	4	6	286	28.9	6.3
**LL23**	B1MMY7	Uncharacterized protein	MAB_1614	36.11	15.96	1	3	3	8	213	22.3	4.88
**LL24**	B1MMY7	Uncharacterized protein	MAB_1614	20.67	15.96	1	3	3	5	213	22.3	4.88
**LL25**	B1MBM0	Uncharacterized protein	MAB_2560	25.26	27.36	1	4	4	7	201	20.5	5.88
**LL25**	B1MG92	Cutinase	MAB_3763	13.46	10.96	1	2	2	4	228	23.5	6.3
**LL26**	B1MBM0	Uncharacterized protein	MAB_2560	13.87	13.43	1	2	2	4	201	20.5	5.88
**LL27**	B1MBM0	Uncharacterized protein	MAB_2560	67.28	37.81	1	6	6	18	201	20.5	5.88
**LL28**	B1MBD4	Uncharacterized protein	MAB_2474	14.46	10.87	1	3	3	4	184	19.1	8.41
**LL29**	B1MFV4	Uncharacterized protein	MAB_0405c	12.74	12.82	1	2	2	4	195	20.2	5.99
**LL29**	B1MDB0	Uncharacterized protein	MAB_3154c	6.6	11.79	1	2	2	2	212	21.7	5.06
**LL30**	B1MBL9	Peptidyl-prolyl cis-trans isomerase	MAB_2559c	13.19	17.86	1	2	2	4	196	19.1	5.06
**LL30**	B1MFV4	Uncharacterized protein	MAB_0405c	11.75	13.33	1	3	3	4	195	20.2	5.99
**LL31**	B1MGD1	Uncharacterized protein	MAB_3801c	19.76	25.7	1	3	3	6	179	18.5	6
**LL32**	B1MGD1	Uncharacterized protein	MAB_3801c	6.1	15.08	1	2	2	2	179	18.5	6
**LL36**	B1MCH9	Uncharacterized protein	MAB_2871c	60.59	22.86	1	4	4	17	140	14.8	5.12
**LL38**	B1MDK0	Soluble secreted antigen MPT53	MAB_3243	5.93	11.8	1	2	2	2	178	19.1	9.57
**LL39**	B1MAH1	Hypothetical low-molecular-weight antigen Mtb12	MAB_2161c	26.23	27.43	1	3	3	7	175	17.3	4.81

**LL39**	B1MDK6	Uncharacterized protein	MAB_3249	10.43	13.61	1	2	2	4	147	16.2	6.58
**LL39**	B1MIH2	Probable aldo/keto reductase	MAB_4120c	3.39	4.26	1	2	2	2	329	35.3	5.11
**LL42**	B1MNM5	D-alanyl-D-alanine dipeptidase	MAB_1843	41.05	35.59	1	6	6	11	222	23.8	6.92
**LL45**	B1MCS9	Uncharacterized protein	MAB_2972	25.05	24.39	1	3	3	7	164	17.6	7.06
**LL47**	B1MMY9	Uncharacterized protein	MAB_1616	14.89	19.44	1	3	3	5	180	20.3	7.97

Table S4: N-terminal signal sequences and probable cleavage sites in the secretory proteins of *M. abscessus* predicted with the in silico analysis of the secreted proteins identified with 2D- GE proteomics.

Serial #	Protein ID	Protein Name	Signal peptide sequence	Cleavage position
1	B1MCR7	Uncharacterized protein	Non-classically secreted protein that lacks SP	-
2	B1MIY6	Uncharacterized protein	Non-classically secreted protein that lacks SP	-
3	B1MMK7	Uncharacterized protein	MTTTRYSVRFAAATASVAALLAGTAAVAMA-DPQP	30
4	B1MEL2	Antigen 85-A	MKLFSKMRGALARQSARRIAVA-ATAV	22
5	B1MEL1	Antigen 85-C	MSVRVKARRVLSALLAAFVMPVSMAAA-MTIN	27
6	B1MEL3	Antigen 85-A//B/C	MKLFSQLRGKTARRLATVAAA-AAVL	21
7	B1MNL7	Immunogenic protein MPT64	MPYLSLRSISASVLVTAASIFGFSGVAGA-APHD	29
8	B1MDW2	Putative secreted hydrolase	MRALRTLSTMIGLAAVVAATAAVPPIASA-EPAA	29
9	B1MMY7	Uncharacterized protein	MTGRITGIVSALVGAVALVLGLFGSMAVATA-SPDT	31
10	B1MFV4	Uncharacterized protein	MKKRLVSAGVIALAAALSGPAALA-AAAP	24
11	B1MBL9	Peptidyl-prolyl cis-trans isomerase	MNLFRVSPSATLAACTASFVMALAACGSDTEANSAPA-DPPS	37
12	B1MCH9	Uncharacterized protein	MTGMGRITAALLATASTATLTLALAPAAEA-APHQ	30
13	B1MIR3	Uncharacterized protein	MLVTLASGVVIGAAAAPMTA-ADPG	20

14	B1MBM0	Uncharacterized protein	MRTIAIRHRAVIGLSAVALITVGCSNG-TSVD	27
15	B1MNM5	D-alanyl-D-alanine dipeptidase	MMKRLILGFGYAALGIGCALGQVAPASA-DPDI	28
16	B1MFV1	Uncharacterized protein	Non-classically secreted protein that lacks SP	-
17	B1MJB0	Uncharacterized protein	MHVGIRTVAVAAVCGLCVGAGVDARA-VRPN	26
18	B1MB96	Uncharacterized protein	MQQPRATSAAKRTHRARVIAATIAVTSLSGAGA-YTLA	33
19	B1MJN7	Aldehyde dehydrogenase family protein	Not predictable as secretory protein	-
20	B1MKW1	Uncharacterized protein	MLVLTRWFRRALVSGVCVAGLLGAGVVPALA-DPGD	31
21	B1MDF1	Elongation factor Ts	Not predictable as secretory protein	-
22	B1MDV5	Uncharacterized protein	MTSVGDKGRRTGPWGRLTAYAAAVAALSGIGLANPI AAHA-DPLI	40
23	B1MEC9	Uncharacterized protein	MRIAASVLAAALCGWAGTAPVVRA-EPSS	24
24	B1MG92	Cutinase	MTLQPNPRPASKLLTAAARAAVVSVAAIMLTTGLAV VIPATASA-ACPN	44
25	B1MBD4	Uncharacterized protein	MKSTYAVLLGIALLLVTPGLASA-DVPR	23
26	B1MDB0	Uncharacterized protein	MIARLITTGLLMAALGLTEAAAAIA-EPID	25
27	B1MGD1	Uncharacterized protein	MDTPMQVSQGRRGISKARVRATGVAALTALALVALPGVA GA-DPEV	41
28	B1MDK0	Soluble secreted antigen MPT53	MNIQFGGRKKFRAQQVRGLTRTVAVVVAVLLVSAGLVRAP SARA-DGLL	44
29	B1MAH1	Hypothetical low-molecular-weight antigen Mtb12	MTGVAMLGLLAAVAVGSAVPTGEPTASS-ASPT	28
30	B1MDK6	Uncharacterized protein	MTSRPQCYVIAAITAAATFCASFFWAQTAAA-EPIV	31
31	B1MIH2	Probable aldo/keto reductase	Not predictable as secretory protein	-
32	B1MCS9	Uncharacterized protein	MRLRPALSLIAALTAVITALAPTASA-LPDR	26
33	B1MMY9	Uncharacterized protein	MKRMLAATALTAALTALGTPAVHA-APNR	24

Potential cleavage sites are indicated by hyphens in amino acid sequences.

Table S5: N-terminal signal sequences and probable cleavage sites in the secreted proteins of *M. abscessus* predicted in silico.

Gene ID	Signal sequences	Amino acid residues
MAB_0046	MNLNVVPEGLTATSAAVEALTARLAAAHAAA-APVI	31
MAB_0063c	MKFSHHLTRRTAGAACVLGAAAVITAPAASA-APDC	31
MAB_0093	MRIAASVLAAALCGWAGTAPVVRA-EPSS	24
MAB_0168c	MRVSKMPVRRQLALAVAATATVMTVSVVDHIKSGQ-AEETA	35
MAB_0169c	MPNRRRRRLSTALSSVAALAVASPFAIALATNMTQAA-APAP	37
MAB_0353	MRTARTVLTSVAAAAFGLTATPAAGA-VPSD	26
MAB_0360	MRSVMGALSVALGVMTPATATA-DPSS	22
MAB_036	MDINGVSAIVTGGASGLGAATARLLAAQGAKVVIA-DVQD	35
MAB_0424	MRYGLPVLIALFGLFAAAIPAAA-APAA	23
MAB_0463c	MAVSRSQWGRRGFLQLGAATAAAGVLA-ACGT	27
MAB_0521	MTEVAVALSGGADSLALAAAAVARA-WSVT	25
MAB_0555	MSPLTRRRLLTSAAGTAAFSAAVSLMPANLQRVLAQP-APRS	37
MAB_0560	MSRDTGRRRPKHALPKSNKRWSVSKTAAA-RTIF	29
MAB_0565c	MKRTLAAATAATAVMSLVLPATAQA-APPK	25
MAB_0576c	MSAATLRDASLNRGGRTLWQGLDLTVEPGEFIAVLGPNGSGK-TSLL	46
MAB_0588c	MHRRHLFKLGAATAGAVMLTACA-DPPD	23
MAB_0620c	MTRWTGLMVAAITLTTGGIAASTAVAPNVACA-CSCV	32
MAB_0684c	MRDLSRRSLLKLGAATGAGAALLGAGAFPAGA-DGAI	32
MAB_0723c	MPTASHRARGALRDPAPQHPRAGKLRILLATALVASLVVTEGA-PTTL	43
MAB_0734	MAGTFIRCIRSACFAAVVVGVAVLQAPPVFA-EPVT	31
MAB_0743c	MRKPLIATIAAVCALALAATGTDFGFA-IFAE	27
MAB_0848c	MLRRLCTATATLALAAAAYPPVAQA-EPEQ	25
MAB_0869c	MSGRHSKPSKASAKAAKVAVAGAGLVGGGLMMAATANA-ATDG	38
MAB_0884c	MKRVWAAVVGGAMLAATQSSCA-ASTQ	22
MAB_0957	MCAALGVGLLSGGAAVAHA-DPAF	19
MAB_1003c	MRISKLFAGRVARGVAGTVVAAAMAGTALVGAPLAGA-SAQS	37
MAB_1026c	MKINVPITAAVAAAAAAAGLALMPTAGA-DTTL	28
MAB_1042c	MYLVTCFAFFMVGGLMALLIRA-ELTT	22
MAB_1161c	MSTGLNRRKLLGAAGVTAAVAGAAGAGVLGGRASA- ASTG	35
MAB_1164	MSSVAVRSLPARFGRIMLVVSISVFLLA-AGCS	28
MAB_1193c	MSILATRRRTIWAALSTGAAGA-AMGT	22
MAB_1261	MTKSGYFATALDAMVVVLPVAAVALA-AVAH	26
MAB_1271c	MNTGMSPRLDRRELLRLGMTAAAAGFLASCTKNTSGA-ATSP	37
MAB_1322	MNKFLCATAVLLSMAGWAPAAAA-EPGN	23
MAB_1323	MATVVMGLAVGLAVPATA-APPP	18
MAB_1363	MVDSGSFRRAFSSFSSFSRLPSPFA-SQSG	25
MAB_1372	MLESSARWFRRKGVALGSSLLTCLTLASLTGCA-RSDD	33
MAB_1543	MKGLSLTAMIAMGAGAAAIAMASPAYA-DDVS	27
MAB_1553c	MDKLRTRVVIGIAAVGMSVGSPFSAGIAQA-NDHT	30
MAB_1616	MKRMLAATALTAALTALGTPAVHA-APNR	24
MAB_1843	MMKRLILGFGYAALGIGCALGQVAPASA-DPDI	28
MAB_1974	MSVGRLLRNHSDRLTPSPLRRAMLAVTAAALVGGVFTGAQTATA-DPNN	44
MAB_2000	MNRQDRSRPVGARRTRRPAAAVSRTAGFA-FRHR	29
MAB_2113	MITNFRRFSAAGILAASAVALAAPAHA-DGEV	27

MAB_2329c	MAVRKRVGAVMALVTAICTFATAPAHA-DPVA	27
MAB_2456	MDPQSRPALMTRRTVLSAGAMLALG-STSL	25
MAB_2462	MNGRAPRRAVLTGAAGIAATAGLFGATGLVRA-AMFD	32
MAB_2546	MCTACEWAPHFAALSAKSTRRTALRAAVALTLAAGAAA-CSAG	38
MAB_2590	MFDRRRFLTLLAGAAAAGASFIEYVSA-AADP	27
MAB_2594	MVPISRRDVLKYAAAIPAAALAGAVSAAAPRANA-ASLG	34
MAB_1511	MVPISRRDVLKYAAAIPAAALAGAVSAAAPRANA-ASLG	34
MAB_2697c	MILGAAVVVATLSGCASSGGQQDAEA-SIAV	26
MAB_2699c	MTSRSLPVPDGLSGMRVDAGLARLLGLSRTAAA-ALAE	30
MAB_2750c	MKFGPGTSAGTAATAAPSLNAAPA-ASTE	24
MAB_2787c	MFGGAPQTAGLFGPRRSASLVVALA-AFLA	25
MAB_2798c	MRLLNIRRYLAVPAVTAFA-AGLA	19
MAB_2799	MLKSLVSLTTVCALALTAPLAFA-DPED	23
MAB_2800	MNDTHSNSSHLSTRGATPMLKSLAAATAVCALAVTAPFALA-DPGD	41
MAB_2805c	MSAVAAVAGVLVASPALSIADPAAET-APKI	26
MAB_2871c	MTGMGRITAALLATASTATLTLALAPAAEA-APHQ	30
MAB_2875	MISRRALLVGGVSAVGVVAAGCSRNGNRRPA-PDEL	31
MAB_2878c	MDSRSPARTGARRIARIGAIVTTVLLAAGLLTSCWTSAA-KTLD	39
MAB_3031c	MTASRLVATMVVAGCCAAIAPA-CTRA	22
MAB_3051	MSRLRDEDARVMRRRHTALVAALALLATLVSA-CGST	32
MAB_3154c	MIARLITTGLLMAALGLTEAAAAIA-EPID	25
MAB_3165c	MLVVTGLVSLLMNAAVTAPATLGHALA-ASPS	27
MAB_3215c	MTVPVVVVALPMGSPGATVSG-SPVH	21
MAB_3264c	MVAVASGAVSLAAAGVALA-DDGD	19
MAB_3305c	MAVMISRRGWLVASAVALSGQAIPA-CSAP	25
MAB_3348	MTSVGDKGRRTGPWGRLTAYAAAVAALSGIGLANPIAAHA-DPLI	40
MAB_3355	MRALRTLSTMIGLAAVVAATAAVPPIASA-EPAA	29
MAB_3390	MQTARLWGAAAISAALVTATVSG-CTEK	23
MAB_3454	MSTRRRISVVLMTSAVIGSSVAVDVAPVAYA-DPCA	31
MAB_3472	MRMLLITCVAVAAGATTFAGQAAA-DPPP	24
MAB_3493	MALTATLMTACTAAVGATAFG-ASLA	21
MAB_3532	MIAPERRTRADIIAAAVIAVVVAVTGTTIWWTSDARA-TVSR	37
MAB_3681	MTTWTPAPDRRPSPRTAVTRLAAATAAGLIVAVLPLCAPPLASA-EPGF 44	
MAB_3749	MTAFPRRTVLRAALLGLAAVPVAA-CGPA	24
MAB_3763	MTLQPNPRPASKLLTAAARAAVVSVAAIMLTTGLAVVIPATASA-ACPN	44
MAB_3765	MTTHSAVLPATSYVRRPGYLLAALLMFASLVAMSQPVPAAA-DPCA	41
MAB_3889	MIKRGLALAAVTALTVAGLNSSQVVA-GYGD	26
MAB_3910	MSDPAPRGPKRPSVISPMVLAPAGTTEA-KALA	28
MAB_3950c	MDATMIILVLLIATALA-FDFT	17
MAB_4037	MSHRLRGLRAGIAATALLASAQVIHPVATARA-ALPP	32
MAB_4061c	MKYPRPHLGDALTRRRALTILGLTVPAVAAA-CTTV	31
MAB_4062c	MRIAIVTLVMWLGLTAAPPATA- DPGP	22
MAB_4185c	MNERVPDSHGLPLRAMVMVLLFLGVIFLLVGFNALG-SDKS	36
MAB_4192	MRRGHVIIAMQTIACLLLLAGSAYL-YTIV	25
MAB_4211c	MLVTLASGVVIGAAAAPMTA-ADPG	20
MAB_4212c	MALFSARGLARGCSLLAIAAVASCCAHPSTPGA-AAPT	33
MAB_4236c	MRFLTVLVCTALTVLAGCAEGA-APGS	22
MAB_4280c	MRGWLRTISALVVIAGLGAAVPAAA-EPKS	25

MAB_4281c	MMMRLFVATLALVAGFSTATAAA-EPTP	23
MAB_4390	MPPGMITRRGLLAAGAALAASTALA-SCAK	25
MAB_4469c	MAELNRRTVLTTGAIAAGA-AAVG	19
MAB_4576c	MRGLLGAFVAGAALLTPVATA-EPHD	21
MAB_4782	MHHSSAPGRHSGSSLVSRRDALRYVLTAPLLAGLPVFAAPQAAA-DGLR	44
MAB_4852c	MKLNRFGAVLTLVASGALVLSACGSDNNASG-SSGS	31
MAB_4903	MTSRRLSTALAGFAIITGAAA-GCGT	21
MAB_4914c	MRGAPVILLSVTVLLSSCTQAIA-GDAT	23
MAB_4915c	MRRGMAAVCIVLSLAGCSRISGGE-PTAP	24
MAB_4924	MTTTRYSVRFAAATASVAALLAGTAAVAMA-DPQP	30

Potential cleavage sites are indicated by hyphens in the amino acid sequences.
